# Glycosylated Hemoglobin Levels in the Third Trimester for Predicting Adverse Pregnancy and Neonatal Outcomes in Women with Pre-Gestational Diabetes: A Multi-Center Retrospective Cohort Study in South Korea

**DOI:** 10.3390/jcm14186389

**Published:** 2025-09-10

**Authors:** Su-Yeon Park, Mi-Ju Kim, Su-Been Hong, Ji-Hee Sung, Hyun-Joo Seol, Joon-Ho Lee, Seung-Chul Kim, Seung-Mi Lee, Se-Jin Lee, Han-Sung Hwang, Gi-Su Lee, Hyun-Soo Park, Soo-Jeong Lee, Sae-Kyung Choi, Ji-Young Kwon, Geum-Joon Cho, Soo-Ran Choi, Hyun-Sun Ko

**Affiliations:** 1 Department of Obstetrics and Gynecology, Medical School, Inha University, Inha University Hospital, Incheon 22332, Republic of Korea; sypark832@inha.ac.kr; 2 Department of Obstetrics and Gynecology, School of Medicine, Kyungpook National University Hospital, Kyngpook National University, Daegu 41944, Republic of Korea; ties1004@naver.com; 3 Department of Obstetrics and Gynecology, Seoul St. Mary’s Hospital, College of Medicine, The Catholic University of Korea, Seoul 06591, Republic of Korea; unihsy@naver.com; 4 Department of Obstetrics and Gynecology, Samsung Medical Center, School of Medicine, Sungkyunkwan University, Seoul 06351, Republic of Korea; jihee.sung@samsung.com; 5 Department of Obstetrics and Gynecology, Korea University Guro Hospital, Korea University, Seoul 08308, Republic of Korea; selhjmd@gmail.com (H.-J.S.); md_cho@hanmail.net (G.-J.C.); 6 Department of Obstetrics and Gynecology, Severance Hospital, College of Medicine, Yonsei University, Seoul 03722, Republic of Korea; jleemd@yuhs.ac; 7 Department of Obstetrics and Gynecology, Pusan National University Hospital, Pusan National University, Busan 49241, Republic of Korea; ksch0127@naver.com; 8 Department of Obstetrics and Gynecology, Seoul National University Hospital, Seoul National University, Seoul 03080, Republic of Korea; lbsm@snu.ac.kr; 9 Department of Obstetrics and Gynecology, Kangwon National University Hospital, Kangwon National University, Chuncheon 24289, Republic of Korea; 23wls@naver.com; 10 Department of Obstetrics and Gynecology, Konkuk University Hospital, Konkuk University, Seoul 05030, Republic of Korea; hwanghs@kuh.ac.kr; 11 Department of Obstetrics and Gynecology, Keimyung University Dongsan Hospital, School of Medicine, Keimyung University, Daegu 41931, Republic of Korea; cllgs315@naver.com; 12 Department of Obstetrics and Gynecology, Dongguk University Hospital, Dongguk University, Goyang 10326, Republic of Korea; 13 Department of Family Medicine, Providence St. Joseph Eureka Hospital, Eureka, CA 95501, USA; 14 Department of Obstetrics and Gynecology, Ulsan University Hospital, College of Medicine, University of Ulsan, Ulsan 44033, Republic of Korea; exsjlee@uuh.ulsan.kr; 15 Department of Obstetrics and Gynecology, Incheon, St. Mary’s Hospital, College of Medicine, The Catholic University of Korea, Incheon 21431, Republic of Korea; obgysk@catholic.ac.kr; 16 Department of Obstetrics and Gynecology, Eunpheong St. Mary’s Hospital, College of Medicine, The Catholic University of Korea, Seoul 03312, Republic of Korea; jiyoungk@catholic.ac.kr

**Keywords:** pre-gestational, diabetes mellitus, glycosylated hemoglobin, third trimester, neonatal outcome, large for gestational age

## Abstract

**Background/Objectives**: The objective of this study is to investigate pregnancy and neonatal outcomes in women with pre-gestational diabetes (PGDM) in the Korean population and compare outcomes according to glycosylated hemoglobin (HbA1c) levels in the third trimester. **Methods**: Singleton pregnant women with PGDM, with follow-up data, and who delivered at 16 Korean tertiary institutions between 2010 and 2023 were included for analysis. Eligible patients were divided into two groups according to HbA1c levels (47.5 mmol/mol, 6.5%) in the third trimester (well-controlled and poorly controlled group). Adverse pregnancy and neonatal outcomes between the two groups were compared. The primary outcome was the composite neonatal adverse outcome and the secondary outcome was pregnancy-related hypertension. **Results**: In 416 pregnancies, the mean HbA1c in the third trimester was 45 mmol/mol (6.26%). Of these, 296 (71.2%) women were included in the well-controlled group and 120 (28.8%) in the poorly controlled group. Between these, the poorly controlled group showed a significantly higher risk of composite neonatal adverse outcome (57.8% vs. 79.2%, *p* < 0.001) and pregnancy-related hypertension (14.5% vs. 24.2%, *p* = 0.022). In multivariate analysis, HbA1c > 6.5% in the third trimester was associated with higher risk of composite neonatal adverse outcome and pregnancy-related hypertension. HbA1c ROC curves for the third trimester that predicted composite neonatal adverse outcomes had an AUC of 0.66; HbA1c of 43.7 mmol/mol (6.15%) had a sensitivity of 52.3% and specificity of 73.5% (*p* < 0.001). **Conclusions**: In PGDM, HbA1c > 47.5 mmol/mol (6.5%) in the third trimester was significantly associated with a higher risk of adverse neonatal and pregnancy outcomes and could be a predictive factor for composite neonatal adverse outcomes and pregnancy-related hypertension. Maintenance of HbA1c levels below 43.7 mmol/mol (6.15%) in the third trimester might decrease the risk of adverse neonatal outcomes.

## 1. Introduction

Pre-gestational diabetes mellitus (PGDM) (either type 1 or 2 diabetes diagnosed before pregnancy) is characterized by hyperglycemia resulting from defects in insulin secretion or action [1]. If DM is identified in the first trimester or early second trimester using the standard diagnostic criteria, it is classified as PGDM [1]. PGDM is observed in approximately 1–2% of pregnancies worldwide, with the rising trend attributed to an overall increase in diabetes mellitus (DM), particularly in urbanized and industrialized population [1,2,3]. Despite its increasing global prevalence, research on PGDM in the South Korean population is limited [4]. This highlights a significant gap in the management of PGDM, demonstrating that the distinct socio-cultural and healthcare contexts that can uniquely influence disease management and outcomes in this group should be considered.

Maternal hyperglycemia during pregnancy is known to increase adverse risks in pregnancy and affect neonatal outcomes [5]. PGDM is associated with adverse neonatal outcomes such as large for gestational age, macrosomia, birth trauma, and neonatal hypoglycemia. The challenge of managing PGDM increases as pregnancy progresses because of increased insulin resistance (a pathophysiological change similar to that in type 2 diabetes), which thus, complicates glycemic control in the later stages of pregnancy [1]. While glycemic control is key to PGDM management, the optimal targets for maternal glycemic control during pregnancy remain controversial. Several studies have demonstrated that elevated HbA1c levels in early pregnancy are associated with increased risk of adverse obstetric outcomes, including preeclampsia, preterm birth, and macrosomia [6]. In addition, longitudinal studies have investigated the relationship between early and late pregnancy HbA1c levels, showing that early HbA1c may predict subsequent glycemic control and outcomes in later trimesters [6]. However, despite these findings, there remains limited evidence focusing specifically on the prognostic value of third-trimester HbA1c for adverse neonatal outcomes, particularly in East Asian populations.

One of the key parameters used to assess glycemic control is glycated hemoglobin (HbA1c), which is widely used as a target for glycemic control in non-pregnant individuals with DM. However, the accuracy of HbA1c during pregnancy is debatable. Owing to physiological changes during pregnancy such as increased red cell production, shortened red cell life span, reduced red cell affinity for glucose, and iron deficiency, the National Institute for Health and Care Excellence (NICE) does not recommend a routine assessment of glycemic control in pregnancy [7].

However, measuring HbA1c to assess glycemic control in pregnancy has been gaining increasing interest. Several studies have reported significant associations between elevated HbA1c levels and increased risk of adverse pregnancy and neonatal outcomes [7,8]. As a result, the International Association of Diabetes and Pregnancy study group (IADPSG) and American Diabetes Association (ADA) guidelines recommend that HbA1c levels > 6.5% (47.5 mmol/mol) can be used as diagnostic criteria for DM in early pregnancy [9].

Despite the growing interest in HbA1c as a prognostic marker during pregnancy, especially in early trimesters, the utility of third-trimester HbA1c remains understudied, particularly in East Asian populations [10,11]. For this reason, we conducted a multi-center retrospective cohort study to assess the association between third-trimester HbA1c levels and adverse pregnancy and neonatal outcomes among South Korea women with PGDM.

## 2. Materials and Methods

This was a multi-center retrospective study of pregnant women who had PGDM and had delivered their babies in 16 Korean tertiary institutions between 2010 and 2023. The study was approved by the Institutional Review Board of all 16centers. The requirement for informed consent was waived due to the retrospective nature of the study.

We included pregnant women who had been diagnosed with PGDM, had follow ups conducted, and had subsequently delivered at the same hospital. PGDM was defined as diabetes diagnosed prior to conception, including both type 1 and 2 diabetes mellitus, as well as cases newly diagnosed during early pregnancy that were considered to have pre-existing diabetes based on clinical assessment. Eligible participants were enrolled consecutively during the study period to minimize selection bias. We excluded women who had chronic hypertension, multiple pregnancy, chromosomal abnormalities, non-viable preterm birth (<24 weeks), or did not have the HbA1c levels recorded in the 3rd trimester, to reduce potential confounding effects on pregnancy outcomes.

All relevant clinical and laboratory data were extracted from institutional electronic databases and medical records by trained research personnel. Pregnancy data collected for this study included maternal age, parity, types of pregnancy, pre-gestational and intrapartum body mass index (BMI), weight gain during pregnancy, the rate of excessive weight gain, co-morbidities, type of DM, duration of DM, use of aspirin, levels of HbA1c and fasting glucose, sonographic findings (estimated fetal weight, bilateral parietal diameter, abdominal circumference, amniotic fluid index, fetal anomaly), admission history during pregnancy, rate of pregnancy-induced hypertension (PIH), diabetic nephropathy, diabetic neuropathy, diabetic retinopathy, diabetic ketoacidosis (DKA) and infection during pregnancy, gestational age at delivery, preterm birth rate before 37 and 34 weeks, mode of delivery, intrauterine fetal death, rate of shoulder dystocia, birth canal trauma (laceration), postpartum hemorrhage, and clinical chorioamnionitis, postpartum endometritis, wound infection, and dehiscence. Excessive weight gain during pregnancy was classified according to pre-pregnancy weight category for South Korean population [12]. The optimal weight gain ranges were as follows: 20.8 kg (16.7–24.7) for underweight (BMI < 18.5 kg/m^2^), 16.6 kg (11.5–21.5) for normal weight (BMI 18.5–22.9 kg/m^2^), 13.1 kg (8.0–17.7) for overweight (BMI 23.0–24.9 kg/m^2^), and 14.4 kg (7.5–21.9) for obese (BMI ≥ 25.0 kg/m^2^). Weight gain exceeding the upper limit of each range was defined as excessive gestational weight gain [12]. Shoulder dystocia was defined as failure of delivering shoulder after downward traction, as identified by medical record or requiring maneuver that assist releasing the impacted shoulders (e.g., McRobert maneuver) in addition to gentle downward traction on the fetal head to affect delivery [13,14]. Birth canal trauma was defined as any events including 3rd or 4th degree laceration, vaginal wall laceration, or urethral or bladder injury. Clinical chorioamnionitis was diagnosed using one or more of the following criteria: maternal fever ≥ 38 °C, maternal or fetal tachycardia, or maternal white blood cell count ≥ 15,000/mm^3^. Neonatal data included birth weight, Apgar score at one and five minutes, neonatal intensive care unit (NICU) admission rate, and neonatal mortality and morbidities (birth trauma, acidosis, polycythemia, hypoglycemia, hypocalcemia, hyperbilirubinemia, respiratory distress syndrome (RDS), sepsis, cardiomyopathy, pulmonary hypertension, seizure, anomaly). Birth trauma was diagnosed using one or more of the following criteria: clavicle or humerus fractures, brachial plexus injury, or hypoxic–ischemic encephalopathy. And hyperbilirubinemia was diagnosed with over 15 mg/dL or need of phototherapy.

**Figure 1:**
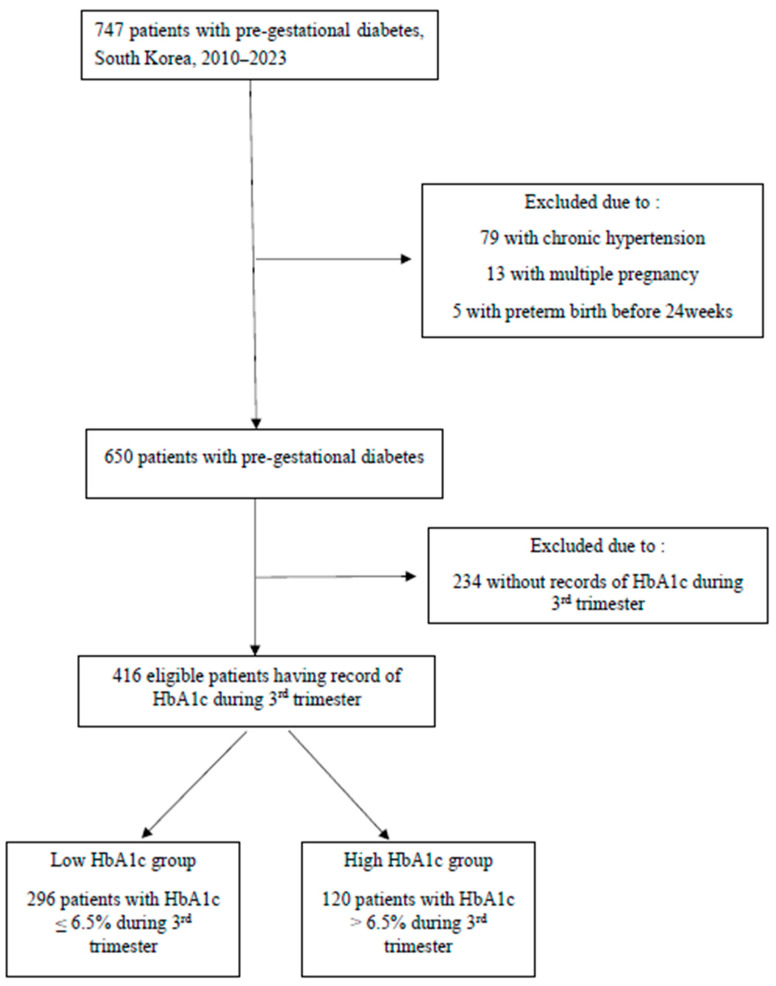
Flowchart of enrollment for study participation.

We divided the participants into two groups according to the HbA1c levels (47.5 mmol/mol, 6.5%) in the 3rd trimester. The HbA1c cut-off of 47.5 mmol/mol (6.5%) was selected based on the diagnostic threshold for diabetes mellitus recommended by the American Diabetes Association and the International Association of Diabetes and Pregnancy Study Groups for early pregnancy, linking this threshold with increased risk of adverse perinatal outcomes [8,9]. Women with HbA1c < 47.5 mmol/mol (6.5%) were categorized as the “Low HbA1c group” and women with HbA1c levels > 47.5 mmol/mol (6.5%) were categorized into the “High HbA1c group” (Figure 1).

We compared the composite neonatal adverse outcomes (primary outcome) and pregnancy outcomes (secondary outcome) between two groups. The composite neonatal adverse outcomes investigated in this study were based on those published in a previous randomized trial and included one of the following events: preterm birth (<37 weeks), intrauterine fetal death (IUFD), large for gestational age (LGA), shoulder dystocia, neonatal birth trauma, RDS, hyperbilirubinema, hypoglycemia, or neonatal death (<7 days) [15]. Large for gestational age (LGA) was defined as birth weight above the 90th percentile for gestational age [16]. The secondary outcome investigated was pregnancy-related hypertension (a composite of preeclampsia, eclampsia, HELLP syndrome, or gestational hypertension). We also evaluated predictive factors associated with the primary and secondary outcomes. Receiver-operating characteristic curve analyses were conducted to determine the HbA1c cut-off value that could predict composite neonatal adverse outcome.

For sample size calculation, assuming an incidence of adverse neonatal outcomes of 60% in the poorly controlled group (HbA1c ≥ 6.5%) and 40% in the well-controlled group (HbA1c < 6.5%), a two-sided test with a 5% alpha level and 80% power required 97 participants per group. After accounting for a 20% loss to follow-up, the total sample size increased to 243 participants. As this was a retrospective cohort study, all eligible cases from participating centers during the study period were included. Descriptive statistics for continuous data were presented as mean (standard deviation) and categorical data as number (percentage). Parametric tests were used to compare the normally distributed data, and comparisons were performed using Student’s *t*-test or the chi-squared test. Univariate and multivariate regression analyses were conducted to determine the risk factors for composite neonatal adverse outcomes and LGA, and the odds ratios (ORs) with 95% confidence intervals (CIs) were calculated. A ROC curve was used to obtain the cut-off value for HbA1c, and the area under the curve (AUC) was used to determine predictive accuracy. All statistical analyses were conducted using IBM SPSS Statistics software (version 26.0; IBM Corp., Armonk, NY, USA). *p* < 0.05 was considered to be statistically significant.

## 3. Results

Our study included a total of 416 singleton pregnancies diagnosed with PGDM from various medical centers. The average maternal age was 34 ± 4 years. Most pregnancies (329/369, 89.2%) were conceived naturally, and 9.5% were conceived through in vitro fertilization with embryo transfer (IVF-ET). The mean pre-pregnancy BMI was 26.2 ± 5.3 kg/m^2^, and the mean intrapartum BMI was 30.9 ± 5.5 kg/m^2^. The mean weight gain during pregnancy was 11.7 ± 6.9 kg, and the rate of excessive weight gain was only 8.2% (33/402). Most patients (342/379, 90.2%) had been diagnosed with type 1 or type 2 diabetes before pregnancy, and 9.8% (37/379) of patients were diagnosed with PGDM during early pregnancy.

We divided the cohort into two groups based on the HbA1c levels (47.5 mmol/mol, 6.5%) in the third trimester. Patients with HbA1c levels ≤ 47.5 mmol/mol (6.5%) were categorized into the “Low HbA1c group,” and those with HbA1c levels > 47.5 mmol/mol (6.5%) were categorized into the “High HbA1c group.” The mean gestational age at which HbA1c was measured between the two groups was not statistically different (33.2 weeks vs. 33.4 weeks, *p* = 0.36). Table 1 presents the maternal characteristics of the two groups. Maternal age, types of pregnancy, pre-pregnancy BMI, and the use of aspirin were not different between the two groups. In the high HbA1c group, multiparous prevalence was higher than in the low HbA1c group (47% vs. 59.2%, *p* = 0.03). Further, the mean intrapartum BMI (30.2 vs. 32.2, *p* = 0.002) and weight gain (11.4 kg vs. 13.9 kg, *p* = 0.001) was higher in the high HbA1c group than in the low HbA1c group. In the low HbA1c group, more women had a previous diagnosis of type 1 or type 2 DM before pregnancy (237/268, 88.4% vs. 105/111, 94.6%) than the high HbA1c group. Moreover, the mean duration of DM was longer in the high HbA1c group than in the low HbA1c group (Table 1).

Further, we compared sonographic findings between the two groups. The sonographic parameters in the second trimester (EFW, BPD, and AC) did not differ between the two groups. However, the mean percentiles of EFW and AC in the third trimester and AFI before delivery were higher in the high HbA1c group than the low HbA1c group (Table 2).

Table 3 shows a comparison of pregnancy outcomes between the two groups. The mean GA at delivery was significantly higher at 37.8 weeks of gestation in the low HbA1c group compared with 37.0 weeks in the high HbA1c group (*p* < 0.001). The rate of preterm birth before 37 weeks was lower in the low HbA1c group than the high HbA1c group (18.6% vs. 30.8%, *p* = 0.009). Mode of delivery, incidence of vacuum-assisted delivery, shoulder dystocia, birth canal trauma, postpartum hemorrhage, chorioamnionitis, and postpartum endometritis did not differ between the two groups. Also, diabetic nephropathy, retinopathy, neuropathy, and ketoacidosis did not differ significantly (Table 3). However, the incidence of pregnancy-related hypertension (14.5%, 24.2%, *p* = 0.022) was higher in the high HbA1c group than the low HbA1c group. The incidence of infection or inflammation during pregnancy and wound infection or dehiscence were also significantly different between the two groups.

Table 4 shows a comparison of the neonatal outcomes between the two groups. The mean birth weight (gram, percentile) was higher in the high HbA1c than the low HbA1c group (3155 g vs. 3452 g, *p* < 0.001). Further, the mean head circumference and height were also higher in the high HbA1c group. In addition, the rate of LGA was significantly higher in the high HbA1c group (49/120, 40.8%) than in the low HbA1c group (47/296, 15.9%) (*p* < 0.001). The rates of Apgar score under 7 at 1 min, NICU admission, and hypoglycemia were higher in the high HbA1c group than the low HbA1c group. However, the rates of Apgar score under 7 at 5 min, neonatal birth trauma, perinatal death < 3 days and 7 days, neonatal polycythemia, acidosis, hypocalcemia, hyperbilirubinemia, RDS, sepsis, cardiomyopathy, pulmonary hypertension, seizure, and anomaly did not differ significantly between the two groups. The mean pH of the umbilical artery was lower in the high HbA1c group than in the low HbA1c group (7.29 ± 0.08 vs. 7.26 ± 0.09, *p* = 0.016). To evaluate the overall burden of adverse neonatal outcomes through a composite index, we defined the composite neonatal adverse outcome as any of the following: preterm birth (<37 weeks), IUFD, LGA, shoulder dystocia, neonatal birth trauma, RDS, hyperbilirubinema, hypoglycemia, or neonatal death (<7 days). Compared to the low HbA1c group, the high HbA1c group showed a significantly higher rate of composite neonatal adverse outcomes (57.8% vs. 79.2%, *p* < 0.001).

To evaluate the factors that were predictive of composite neonatal adverse outcome, we performed bivariable and multivariate logistic regression analyses. HbA1c levels > 6.5% (47.5 mmol/mol) in the third trimester (adjusted OR [aOR] 2.52; 95% CI, 1.45─4.39) and pregnancy-related hypertension (aOR 2.36; 95% CI1.13─4.94) were associated with a significantly increased risk of composite neonatal adverse outcomes (Table 5).

**Figure 2:**
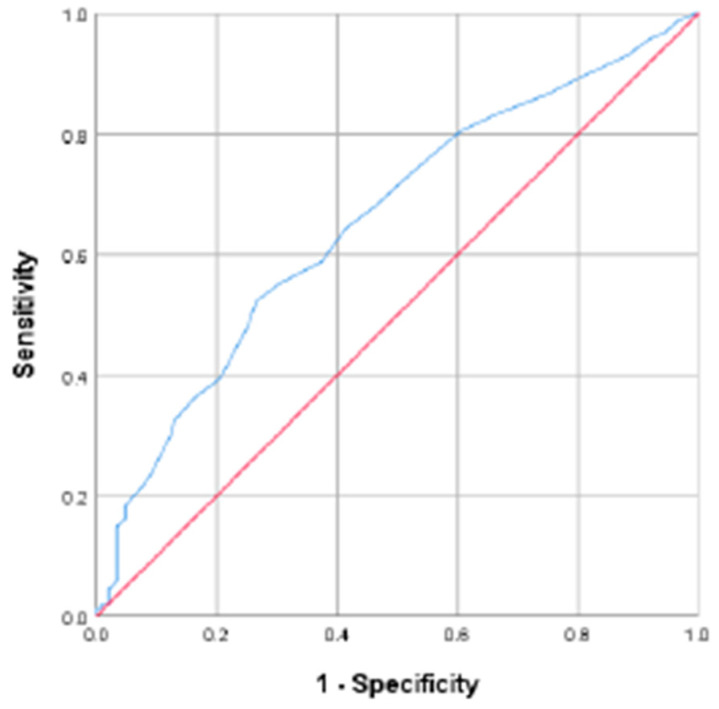
ROC curve (Blue curve) for HbA1c during third trimester and neonatal composite outcomes (AUC, 0.655; cut-off value, 43.7 mmol/mol; sensitivity, 52.3%; specificity, 73.5%) (Red curve, no discrimination corresponding random classification, which means AUC = 0.5).

The ROC curve for HbA1c levels in the third trimester is presented in Figure 2. The AUC was 0.655 and the optimal cut-off value of HbA1c was 43.7 mmol/mol (6.15%) (sensitivity = 52.3%, specificity = 73.5%) (Figure 2).

Logistic regression analyses were performed to evaluate the factors that contributed to pregnancy-related hypertension. Table 6 shows the results for the bivariable and multivariable regression analyses of the predictive factors that were associated with pregnancy-related hypertension. In the bivariable analysis, HbA1c > 47.5 mmol/mol (6.5%) in the third trimester (OR, 1.88; CI, 1.11–3.18), multiparity (OR, 0.57; CI, 0.34–0.95), and pre-pregnancy overweight or obesity (BMI > 23) (OR, 2.31; CI, 1.06–5.05) were associated with pregnancy-related hypertension. Advanced maternal age intrapartum obesity (BMI > 25) and excessive gestational weight gain were not associated with it. In the multivariable analysis, HbA1c 6.5% in the third trimester increased the risk of pregnancy-related hypertension (aOR, 1.92; 95% CI, 1.02–3.61) and multiparous pregnancy decreased the risk (aOR, 0.50; 95% CI, 0.27–0.93) (Table 6).

We also performed a logistic regression analysis and ROC curve analysis to assess the factors that contributed to LGA. Supplementary S1 shows the results of the bivariable and multivariable analyses. HbA1c levels > 6.5% in the third trimester and excessive weight gain were associated with increased risk of LGA (aOR, 3.98; 95% CI, 2.26–7.02, and aOR 2.80; 95% CI, 1.21–6.45, respectively), and advanced maternal age (>35 yrs) and duration of DM were associated with decreased risk of LGA (aOR, 0.52; 95% CI, 0.29–0.92 and aOR 0.51; 95% CI, 0.28–0.94, respectively) (Supplementary S1). The optimal cut-off value for HbA1c that was predictive of LGA was 6.15% (43.7 mmol/mol) (AUC = 0.722, Sn = 75%, Sp = 67.2%) (Supplementary S2).

## 4. Discussion

Our study demonstrated that high HbA1c diabetes (as indicated by HbA1 level > 47.5 mmol/mol, 6.5%) in the third trimester was significantly associated with adverse neonatal and pregnancy outcomes in South Korean pregnant women. Moreover, low HbA1c levels (<43.7 mmol/mol, 6.15%) in the third trimester were associated with reduced risk of composite neonatal adverse outcomes, LGA, and pregnancy-related hypertension.

While type 1 and 2 diabetes differ in etiology and management, both ultimately result in maternal hyperglycemia, which is the key determinant of adverse pregnancy and neonatal outcomes [5,6,7,8]. The impact of hyperglycemia on fetal development such as LGA neonates, preterm delivery, and neonatal hypoglycemia has been shown to be more closely associated with the level of glycemic control rather than the diabetes subtypes. Moreover, several previous studies assessing pregnancy outcomes in pre-gestational diabetes have pooled type 1 and 2 diabetes to focus on glycemic control indicators such as HbA1c [17,18]. In our cohort, the primary objective was to evaluate whether third-trimester HbA1c serves as a predictor for poor outcomes. Therefore, we analyzed type 1 and 2 diabetes patients together as a single PGDM group stratified by HbA1c level.

These findings are consistent with other studies conducted in different populations. A study published in the diabetes care field found that HbA1c between 6.5 and 6.9% at 34 weeks of gestation was significantly associated with increased risk of adverse pregnancy outcomes like preterm delivery, LGA, preeclampsia, the need for a neonatal glucose infusion, and composite adverse outcomes highlighting the need to maintain glycemic controls during the later stages of pregnancy to mitigate those risks [16]. That study also showed a risk of composite adverse neonatal outcome between an HbA1c 6.5% and 6.9%, but the confidence interval included unity (aOR, 2.7, CI, 1.0–7.5). Another study reported that women with PGDM who achieved an HbA1c target below 6.5% during the late stages of pregnancy had significantly reduced rates of adverse outcomes, irrespective of their early pregnancy glycemic control [18].

Glycemic control during the third trimester is crucial because it plays a key role in determining pregnancy and neonatal outcomes through its effects on maternal and fetal physiology. As the pregnancy progresses into the third trimester, placental hormones such as human placental lactogen, cortisol, and progesterone can cause a substantial increase in insulin resistance [1]. Effective glycemic control during this period is therefore critical to mitigate the antagonistic effects of insulin and to maintain metabolic homeostasis.

Moreover, glycemic control is critical for fetal growth and development. Previous research has shown that poorly controlled diabetes is linked to adverse outcomes such as macrosomia, which can lead to birth complications such as shoulder dystocia, birth trauma, and cesarean section. The HAPO study revealed that hyperglycemia directly correlates with an increased risk of macrosomia, neonatal hypoglycemia, and high cord blood C-peptide levels, which are indicative of fetal hyperinsulinemia [5]. ADA also reported that pregnant women with HbA1c < 6% in the second and third trimesters have the lowest risk of LGA infants [9]. These findings are remarkably similar to our results, which showed an increased risk of LGA with HbA1c levels > 6.5%. Our study also showed that an HbA1c cut-off value of 6.15% was predictive of LGA. In this study, advanced maternal age (>35 yrs) and duration of DM were associated with decreased risk of LGA. We speculate that cardiovascular changes in maternal aging and long duration of DM may affect placenta and maternal–fetal circulation [19,20].

Several markers can measure and identify glycemic control. Of those, HbA1c was chosen for several reasons. HbA1c levels are not influenced by short-term lifestyle changes such as diet or exercise, which can affect fasting and postprandial glucose tests. Despite the occurrence of normal physiological changes such as increased red blood cell turnover, the stability of HbA1c makes it a more reliable indicator of long-term glycemic exposure and control, allowing for better assessment of maternal glycemic control during pregnancy. Second, HbA1c measurement is very simple and convenient and requires only a single blood draw and can be measured in fasted or fed states. This convenience of blood sampling can lead to higher patient compliance and easier integration into routine prenatal care. Third, several researchers have reported the predictive value of HbA1c for adverse pregnancy and neonatal outcomes [17,18,19,20,21]. Hence, the use of HbA1c measurements is recommended by IADPSG and ADA guidelines, which recommended the use of HbA1c > 6.5% for diagnosis and for adjusting therapeutic intervention during pregnancy [9,10]. Due to increased red blood cell turnover, HbA1c is slightly lower during pregnancy in people with and without diabetes. ADA recommends an HbA1c target of <6% during pregnancy, if it can be achieved without significant hypoglycemia. But the target can be increased to <7% if necessary to prevent hypoglycemia [9].

In our study, we used Korean optimal gestational weight gain (GWG), which is higher and wider than the Institute of Medicine (IOM) guidelines [12]. This discrepancy is particulary evident in the overweight and obese categories, where the Korean data suggest higher optimal GWG ranges, which appears couterintuitive. One possible reason is that the Korean population has a higher proportion of underweight women and a lower prevalence of maternal obesity compared to Western populations, which shifts the optimal GWG upward to ensure adequate fetal growth. Second, the prevalence of LGA is relatively low and the risk of SGA is higher in the Korean population; therefore, additional weight gain may be protective agaist fetal growth restriction without markedly increasing the risk of macrosomia. Moreover, methodological differences including the use of Asian-specific BMI classification and continuous modeling of GWG also contributed to broader ranges [12].

Our study has some limitations which should be acknowledged. First, our sample could be prone to selection bias owing to the retrospective nature of the study and the exclusion of patients with incomplete data. Second, the sample size (*n* = 416) may not be large enough to generalize to the entire South Korean population, which could cause a type II error. Third, potential confounding factors such as HbA1c level during early pregnancy, the socioeconomic status of the participant, or pregnancy-related anemia could have influenced the outcomes. Furthermore, detailed characteristics of multiple centers were not consistently available and therefore could not be adjusted for in the multivarite analysis. This could introduce potential confounding effects related to center-specific practices or patient population. In addition, the association between HbA1c level and pregnancy-related hypertension should be interpreted with caution due to lack of the exact timing of pregnancy-related hypertension. Moreover, data on baseline glycemic control (e.g., fasting glucose or HbA1c in early or mid-pregnancy) and the details of glycemic medications (type, dosage, duration) were not consistently avaiable, limiting our ability to fully assess the impact of early glycemic status or treatment patterns on pregnancy outcomes.

The strengths of the study are that it was the largest multi-center retrospective cohort study to evaluate clincial characteristics of PGDM including type 1 and 2 and cases with diagnosis during early pregnancy in the South Korean population. Further, it compared pregnancy and neonatal outcomes according to HbA1c levels in the third trimester. Previous studies in the South Korean population investigated a population of only type 1 or 2 DM patients [20,21]. Additionally, those studies were small-sized single-center studies which compared pregnancy outcomes between type 1 DM, type 2 DM, or controls [22,23]. Moreover, in our study, we also used ROC analysis to determine an HbA1c value of 6.15% as an optimal cut-off value for predicting pregnancy and neonatal outcomes.

Further studies including a large randomized study, prospective cohort studies, subgroup analysis by diabetes type, and the integration of continuous glucose monitoring data are needed to compare the pregnancy and neonatal outcomes of PGDM according to glycemic control in the third trimester and determine the confounding factors associated with pregnancy and neonatal outcomes.

## 5. Conclusions

An increased risk of pregnancy-related and neonatal adverse outcomes in women with PGDM was observed if HbA1c levels in the third trimester were higher than 6.5% (47.5 mmol/mol). Also maintenance of HbA1c under 6.15% (43.7 mmol/mol) during the third trimester might decrease the risk of composite neonatal adverse outcomes, as well as pregnancy-related hypertension and LGA.

## Figures and Tables

**Table 1:** Maternal characteristics according to HbA1c levels in third trimester.

	Low HbA1c Group (*n* = 296)	High HbA1c Group (*n* = 133)	*p*-Value
Maternal age (years)	34 (4)	34 (4)	0.406
Parity			0.030 *
Primiparous	157 (53%)	49 (40.8%)	
Multiparous	139 (47%)	71 (59.2%)	
Types of pregnancy			0.142
Natural pregnancy	226 (86.3%)	103 (94.5%)	
COH + IUI	3 (1.1%)	1 (0.9%)	
IVF-ET	31 (11.8%)	4 (3.7%)	
Others	1 (0.4%)	0 (0%)	
Pre-pregnancy BMI (kg/m^2^)	26.0 (4.9)	26.9 (6.4)	0.187
Intrapartum BMI (kg/m^2^)	30.2 (5.8)	32.2 (5.9)	0.002 *
Weight gain during pregnancy (kg)	11.4 (6.4)	13.9 (7.0)	0.001 *
Excessive weight gain	19/294 (6.5%)	14/108 (13.0%)	0.041 *
Co-morbidities	88/294 (29.9%)	37/119 (31.1%)	0.814
Types of DM			0.045 *
Type 1 or 2	237/268 (88.4%)	105/111 (94.6%)	
Diagnosed during early pregnancy	31/268 (11.6%)	6/111 (5.4%)	
Duration of DM (years)	5.2 (6.1)	6.3 (7.1)	0.001 *
Use of Aspirin	88 (29.9%)	37 (31.1%)	0.814

Data are expressed as means (standard deviations) and numbers (percentages). * means statistical significance. COH + IUI, controlled ovarian hyperstimulation with intrauterine insemination; IVF-ET, in vitro fertilization and embryo transfer; BMI, body mass index; DM, diabetes mellitus.

**Table 2:** Sonographic findings according to HbA1c levels in third trimester.

	Low HbA1c Group (*n* = 296)	High HbA1c Group (*n* = 133)	*p*-Value
EFW (percentile) at 2nd trimester	53.5 (24.7)	60.0 (22.6)	0.045
BPD (percentile)at 2nd trimester	51 (25.3)	56.3 (25.3)	0.308
AC (percentile) at 2nd trimester	49.5 (25.2)	53.6 (26.9)	0.379
EFW (percentile) at 3rd trimester	53.1 (27.6)	69.1 (26.7)	<0.001 *
BPD (percentile) at 3rd trimester	48.3 (30.5)	54.5 (30.5)	0.187
AC (percentile) at 3rd trimester	48.1 (32.4)	75.4 (27.3)	<0.001 *
AFI (cm) before delivery	13 (5)	15 (6)	0.001 *
Polyhydramnios	10/263 (3.8%)	8/104 (7.7%)	0.176
Oligohydramnios	11/263 (4.2%)	2/104 (1.9%)	0.365

Data are expressed as means (standard deviations) and numbers (percentages). * means statistical significance. EFW, estimated fetal weight; BPD, biparietal diameter; AC, abdominal circumference; AFI, amniotic fluid index.

**Table 3:** Pregnancy outcomes according to HbA1c levels in third trimester.

	Low HbA1c Group (*n* = 296)	High HbA1c Group (*n* = 133)	*p*-Value
GA at delivery (weeks)	37.8 (1.5)	37.0 (2.0)	<0.001 *
Preterm delivery < 37 weeks	55 (18.6%)	37 (30.8%)	0.009 *
Preterm delivery < 34 weeks	5 (1.7%)	6 (5.0%)	0.086
Mode of delivery			0.198
Vaginal delivery	72 (24.3%)	22 (18.3%)	
Cesarean section	224 (75.7%)	98 (81.7%)	
Vacuum-assisted delivery	8/72 (11.1%)	4/22 (18.2%)	0.466
Shoulder dystocia	8/72 (11.1%)	6/22 (27.3%)	0.086
Birth canal trauma	2/72 (2.8%)	0/22 (0%)	0.585
Postpartum hemorrhage	11 (3.7%)	6 (5.0%)	0.587
Chorioamnionitis	3 (1.0%)	1 (0.8%)	0.672
Postpartum endometritis	0 (0%)	0 (0%)	1
Wound infection/dehiscence	0 (0%)	3 (2.5%)	0.024 *
History of admission during pregnancy	102 (34.5%)	54 (45.0%)	0.029 *
^†^ Pregnancy-related hypertension	43 (14.5%)	29 (24.2%)	0.022 *
Diabetic nephropathy	4/295 (1.4%)	4/120 (3.3%)	0.236
Diabetic retinopathy	12/295 (4.1%)	6/120 (5.0%)	0.791
Diabetic neuropathy	2/295 (0.7%)	3/120 (2.5%)	0.148
Diabetic ketoacidosis	1/295 (0.3%)	2/120 (1.7%)	0.202
Infection/inflammation	8/295 (2.7%)	11/120 (9.2%)	0.008 *

Data are expressed as means (standard deviations) and numbers (percentages). * means statistical significance. ^†^ Pregnancy-related hypertension: any of preeclampsia, eclampsia, HELLP syndrome, or gestational hypertension. GA, gestational age.

**Table 4:** Neonatal outcomes according to HbA1c levels in third trimester.

	Low HbA1c Group (*n* = 296)	High HbA1c Group (*n* = 133)	*p*-Value
Neonatal birth weight (gram)	3155 (614)	3452 (732)	<0.001 *
LGA	47 (15.9%)	49 (40.8%)	<0.001 *
HC (percentile)	50.5 (35.4)	62.0 (26.9)	0.017 *
Height (percentile)	52.7 (24.6)	61.4 (23.7)	0.007 *
Apgar score < 7 at 1 min	59 (19.9%)	37 (30.8%)	0.021 *
Apgar score < 7 at 5 min	9 (3.0%)	5 (4.2%)	0.557
Neonatal birth trauma	4/72 (5.6%)	1/22 (4.5%)	0.107
NICU admission	102/284 (35.9%)	62/118 (52.5%)	0.003 *
Duration of NICU admission (days)	10.6 (12.2)	11.9 (19.5)	0.591
Perinatal death (<3 days)	1/284 (0.4%)	1/118 (0.8%)	0.501
Perinatal death (<7 days)	0/284 (0%)	0/118 (0%)	1
Neonatal polycythemia	4/185 (2.2%)	3/85 (3.5%)	0.682
pH < 7.1	1/197 (0.5%)	2/89 (2.2%)	0.229
Hypoglycemia	19/276 (6.9%)	18/114 (15.8%)	0.012 *
Hypocalcemia	25/203 (12.3%)	13/92 (14.1%)	0.709
Hyperbilirubinemia	71/243 (29.2%)	33/107 (30.8%)	0.800
RDS	25/283 (8.8%)	14/118 (11.9%)	0.359
Sepsis	6/283 (2.1%)	3/118 (2.5%)	0.726
Cardiomyopathy	5/283 (1.8%)	1/118 (0.8%)	0.675
Pulmonary hypertension	1/283 (0.4%)	2/118 (1.7%)	0.208
Seizure	3/283 (1.1%)	1/118 (0.98%)	0.666
Anomaly	43/284 (15.1%)	26/118 (22.0%)	0.110
^†^ Composite neonatal adverse outcomes	167/289 (57.8.%)	95/120 (79.2%)	<0.001 *

Data are expressed as means (standard deviations) and numbers (percentages). * means statistical significance. ^†^ Composite neonatal adverse outcome: any of preterm birth (<37 weeks), IUFD, LGA, shoulder dystocia, birth trauma, RDS, neonatal death, hyperbilirubinemia (phototherapy), NICU admission, hypoglycemia. LGA, large for gestational age; HC, head circumference; NICU, neonatal intensive care unit; RDS, respiratory distress syndrome.

**Table 5:** Logistic regression analyses of factors associated with neonatal composite adverse outcomes.

	Odds Ratio (95% CI)	*p*-Value	aOR (95% CI) ^†^	*p*-Value
HbA1c > 47.5 mmol/mol (6.5%) at 3rd trimester	2.78 (1.69–4.57)	<0.001 *	2.52 (1.45–4.39)	0.001 *
Advanced maternal age (>35 years)	0.86 (0.57–1.29)	0.463	0.96 (0.60–1.52)	0.859
Multiparous	1.02 (0.68–1.53)	0.922	0.99 (0.63–1.58)	0.981
Pre-pregnancy overweight/obesity (BMI > 23 kg/m^2^)	1.68 (1.04–2.71)	0.035	1.72 (0.98–2.99)	0.058
Intrapartum obesity (BMI > 25 kg/m^2^)	1.23 (0.69–2.20)	0.484	0.76 (0.39–1.50)	0.424
Excessive gestational weight gain (>Optimal weight gain range)	1.64 (0.73–3.60)	0.232	1.63 (0.68–3.90)	0.276
Pregnancy-related hypertension	2.24 (2.27–4.31)	0.006 *	2.36 (1.13–4.94)	0.022 *
DM type 1 or 2	1.88 (0.93–3.77)	0.078	2.20 (0.95–5.10)	0.067
Duration of DM (>5 years)	1.36 (0.88–2.10)	0.164	1.20 (0.74–1.96)	0.459

* means statistical significance. ^†^ Adjustment was made for high HbA1c (>6.5%), advanced maternal age, multiparous, pre-pregnancy overweight/obesity, intrapartum obesity, excessive gestational weight gain, pregnancy-related hypertension, DM type 1 or 2, and duration of DM. BMI, body mass index; DM, diabetes mellitus; CI, confidence interval; aOR, adjusted odds ratio.

**Table 6:** Logistic regression analyses of factors associated with pregnancy-related hypertension.

	Odds Ratio (95% CI)	*p*-Value	aOR (95% CI) ^†^	*p*-Value
HbA1c > 47.5 mmol/mol (6.5%) at 3rd trimester	1.88 (1.11–3.18)	0.020 *	1.92 (1.02–3.61)	0.043 *
Advanced maternal age (>35 years)	1.03 (0.62–1.71)	0.921	0.93 (0.50–1.71)	0.806
Multiparous	0.57 (0.34–0.95)	0.032 *	0.50 (0.27–0.93)	0.027 *
Pre-pregnancy overweight/obesity (BMI > 23)	2.31 (1.06–5.05)	0.035 *	2.45 (1.00–6.03)	0.051
Intrapartum obesity (BMI > 25)	1.25 (0.56–2.77)	0.588	0.84 (0.34–2.07)	0.706
Excessive gestational weight gain (>Optimal weight gain range)	1.39 (0.58–3.34)	0.466	1.53 (0.59–3.95)	0.385
DM type 1 or 2	0.82 (0.34–1.96)	0.658	0.65 (0.22–1.94)	0.436
Duration of DM (>5 years)	1.21 (0.70–2.10)	0.496	1.60 (0.85–3.01)	0.146

* means statistical significance. ^†^ Adjustment was made for high HbA1c (>6.5%), advanced maternal age, multiparous, pre-pregnancy overweight/obesity, intrapartum obesity, excessive gestational weight gain, DM type 1 or 2, and duration of DM. BMI, body mass index; DM, diabetes mellitus; CI, confidence interval; aOR, adjusted odds ratio.

## Data Availability

The datasets used and/or analyzed during the current study are available from the corresponding author on reasonable request.

## Supplementary Materials

The following supporting information can be downloaded at: https://www.mdpi.com/article/10.3390/jcm14186389/s1, Supplementary S1: Logistic regression analyses of factors associated with large for gestational age; Supplementary S2: Receiver-operating characteristic curve for HbA1c for the third trimester and large for gestational age (AUC, 0.722; Cut-off value, 6.15%; Sensitivity, 75%; Specificity, 67.2%).

## Abbreviations

The following abbreviations are used in this manuscript:

PGDM	Pre-gestational diabetes mellitus
HbA1c	Glycosylated hemoglobin
LGA	Large for gestational age
BMI	Body mass index
DKA	Diabetic ketoacidosis
NICU	Neonatal intensive care unit
RDS	Respiratory distress syndrome
OR	Odds ratio
CI	Confidence interval
