# Supplementary material for: Glycosylated Hemoglobin Levels in the Third Trimester for Predicting Adverse Pregnancy and Neonatal Outcomes in Women with Pre-Gestational Diabetes: A Multi-Center Retrospective Cohort Study in South Korea
Source: J Clin Med. 2025 Sep 10;14(18):6389. doi: 10.3390/jcm14186389 (PMC12470566; doi:10.3390/jcm14186389)

Supplementary S1. Logistic regression analyses of factors associated with large for gestational age (LGA)

	Odd ratio (95% CI)	*p*-value	aOR (95% CI) †	*p*-value
HbA1c>47.5mmol/mol (6.5%) at 3rd trimester	3.66 (2.26-5.90)	<0.001*	3.98 (2.26-7.02)	<0.001*
Advanced maternal age (>35 years)	0.71 (0.45-1.13)	0.153	0.52 (0.29-0.92)	0.025*
Multiparous	1.35 (0.85-2.14)	0.198	1.56 (0.88-2.75)	0.129
Pre-pregnancy overweight/obesity (BMI >23)	1.53 (0.83-2.82)	0.176	1.45 (0.69-3.07)	0.328
Intrapartum obesity (BMI>25)	2.25 (0.98-5.16)	0.055	1.23 (0.49-3.10)	0.667
Excessive gestational weight gain (> Optimal weight gain range)	2.47 (1.17-5.18)	0.017*	2.80 (1.21-6.45)	0.016*
DM type 1 or 2	1.68 (0.68-4.17)	0.262	4.35 (0.96-19.62)	0.056
Duration of DM (>5 years)	0.73 (0.44-1.20)	0.214	0.51 (0.28-0.94)	0.029*

*, which means statistical significance.

†Adjustment was made for high HbA1c (>47.5mmol/mol, 6.5%), advanced maternal age, multiparous, pre-pregnancy overweight/obesity, intrapartum obesity, excessive gestational weight gain, DM type 1 or 2 and duration of DM and duration of DM.

BMI, body mass index; DM, diabetes mellitus; CI, confidence interval; aOR, adjusted odd ratio.

Supplementary S2. Receiver-operating characteristic curve for HbA1c for the third trimester and large for gestational age (AUC, 0.722; Cut-off value, 43.7mmol/mol, 6.15%; Sensitivity, 75%; Specificity, 67.2%)

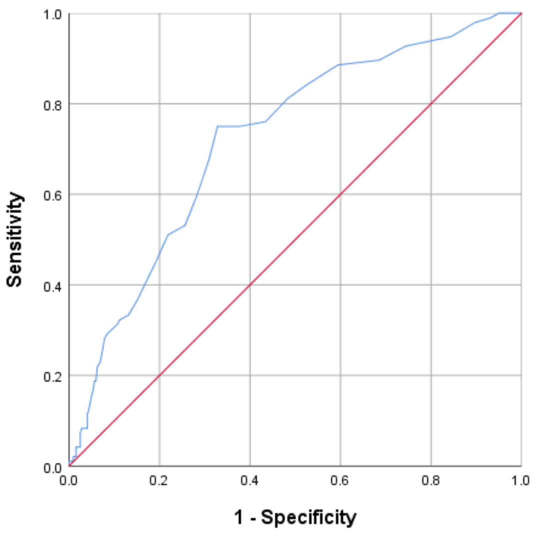

Supplement: Supplementary file 1 [file jcm-14-06389-s001.zip › jcm-3779573-supplementary.pdf]
